# Impact of dengue virus (DENV) co-infection on clinical manifestations, disease severity and laboratory parameters

**DOI:** 10.1186/s12879-016-1731-8

**Published:** 2016-08-11

**Authors:** Amreeta Dhanoa, Sharifah Syed Hassan, Chin Fang Ngim, Chun Fatt Lau, Teik Seng Chan, Nur Amelia Azreen Adnan, Wilhelm Wei Han Eng, Han Ming Gan, Ganeswrie Rajasekaram

**Affiliations:** 1 Jeffrey Cheah School of Medicine and Health Sciences, Monash University Malaysia, Bandar Sunway, 47500 Selangor Malaysia; 2 Clinical School Johor Bahru, Jeffrey Cheah School of Medicine and Health Sciences, Monash University Malaysia, 80100 Johor Bahru, Johor Malaysia; 3 Monash University Malaysia Genomics Facility, Bandar Sunway, 47500 Selangor Malaysia; 4 School of Science, Monash University Malaysia, Bandar Sunway, 47500 Selangor Malaysia; 5 Department of Pathology, Hospital Sultanah Aminah Johor Bahru, 80100 Johor Bahru, Johor Malaysia

**Keywords:** Dengue virus, DENV, Serotype, Co-infection, RT-PCR, Clinical manifestations

## Abstract

**Background:**

The co-circulation of 4 DENV serotypes in geographically expanding area, has resulted in increasing occurrence of DENV co-infections. However, studies assessing the clinical impact of DENV co-infections have been scarce and have involved small number of patients. This study explores the impact of DENV co-infection on clinical manifestations and laboratory parameters.

**Methods:**

This retrospective study involved consecutive hospitalized patients with non-structural protein 1 (NS1) antigen positivity during an outbreak (Jan to April 2014). Multiplex RT-PCR was performed directly on NS1 positive serum samples to detect and determine the DENV serotypes. All PCR-positive serum samples were inoculated onto C6/36 cells. Multiplex PCR was repeated on the supernatant of the first blind passage of the serum-infected cells. Random samples of supernatant from the first passage of C6/36 infected cells were subjected to whole genome sequencing. Clinical and laboratory variables were compared between patients with and without DENV co-infections.

**Results:**

Of the 290 NS1 positive serum samples, 280 were PCR positive for DENV. Medical notes of 262 patients were available for analysis. All 4 DENV serotypes were identified. Of the 262 patients, forty patients (15.3 %) had DENV co-infections: DENV-1/DENV-2(85 %), DENV-1/DENV-3 (12.5 %) and DENV-2/DENV-3 (2.5 %). Another 222 patients (84.7 %) were infected with single DENV serotype (mono-infection), with DENV- 1 (76.6 %) and DENV- 2 (19.8 %) predominating. Secondary dengue infections occurred in 31.3 % patients. Whole genome sequences of random samples representing DENV-1 and DENV-2 showed heterogeneity amongst the DENVs.

Multivariate analysis revealed that pleural effusion and the presence of warning signs were significantly higher in the co-infected group, both in the overall and subgroup analysis. Diarrhoea was negatively associated with co-infection. Additionally, DENV-2 co-infected patients had higher frequency of patients with severe thrombocytopenia (platelet count < 50,000/mm^3^), whereas DENV-2 mono-infections presented more commonly with myalgia. Elevated creatinine levels were more frequent amongst the co-infected patients in univariate analysis. Haemoconcentration and haemorrhagic manifestations were not higher amongst the co-infected patients. Serotypes associated with severe dengue were: DENV-1 (*n* = 9), DENV-2 (*n* = 1), DENV-3 (*n* = 1) in mono-infected patients and DENV-1/DENV-2 (*n* = 5) and DENV-1/DENV-3 (*n* = 1) amongst the co-infected patients.

**Conclusion:**

DENV co-infections are not uncommon in a hyperendemic region and co-infected patients are skewed towards more severe clinical manifestations compared to mono-infected patients.

## Background

Dengue virus (DENV) infection is a global health threat, with approximately half of the world’s population at risk of being infected and 0.5 million people requiring hospitalization each year [1]. It is amongst the most important vector-borne viral disease of humans. A recent estimate suggests that there are 96 million apparent DENV infections globally per year with Asian countries bearing 70 % of this burden [2], making this region an epicentre of dengue activity. DENV infections can lead to a wide range of clinical manifestations, ranging from mild fever to potentially fatal dengue shock syndrome. Previously classified as dengue fever (DF), dengue haemorrhagic fever (DHF) and dengue shock syndrome (DSS) [3], World Health Organization (WHO) dengue classification 2009 classifies dengue as dengue with or without warning signs and severe dengue [4].

DENV, a positive-stranded RNA virus of the *Flaviviridae* family has 4 distinct serotypes (DENV-1, DENV-2, DENV-3 and DENV-4). DENV is transmitted to humans by the *Aedes* mosquito, principally the *Aedes aegypti* mosquito [5]. Dengue has now become hyper-endemic in many countries including Malaysia with all four DENV serotypes co-circulating, with fluctuations of the dominant serotypes over time and location [6]. To date, studies describing the wide arrays of clinical characteristics associated with different DENV serotypes have been widely described. Some studies have suggested that DENV-2 leads to more severe disease, whereas DENV-1 is responsible for milder illness [7]. The co-circulation of multiple DENV serotypes in the same region, invariably facilitates the occurrence of co-infections with rates ranging from 5 % to 30 % [8–15] to as high as 40 % to 50 % [16, 17]. This phenomenon has heightened the importance of understanding the role of co-infections in the clinical outcome of disease. However, although DENV co-circulation is reasonably common in tropical countries, very little emphasis has been placed so far on co-infections. Moreover, only a handful of studies have actually explored the clinical implications of co-infections [8, 10, 12, 14, 17]. Vast majority of these studies were descriptive in nature and the numbers of co-infections were rather small to reach valid statistical conclusions.

Thus, there still remains an unresolved question as to whether the clinical manifestations of dengue vary between co-infected and mono-infected patients. In anticipation of an increasing number of co-infections, exploring the various characteristics and disease severity associated with co-infections, will undoubtedly enhance our understanding of the dynamics and impact of these infections. To the best of our knowledge, this study has the largest number of DENV co-infected patients, allowing more reliable interpretation of findings.

This study aims to determine the clinical and laboratory characteristics amongst patients hospitalized with DENV infections, specifically exploring the effects of DENV co-infections on these patients.

## Methods

### Patients and setting

This research was conducted at Hospital Sultanah Aminah Johor Bahru (HSAJB), a 989-bedded hospital that serves as the main tertiary referral centre of Southern Malaysia, with its patient population reflecting the larger community in Malaysia. The period of study was from January to April 2014, coinciding with one of the peaks of DENV outbreaks. During the study period, all hospitalized patients with a positive non-structural protein 1 (NS1) antigen were identified from the microbiology laboratory database, HSAJB. The initial NS1 testing was done at the microbiology laboratory of HSAJB, using a commercially available rapid dengue diagnostic kit; SD BIOLINE Dengue Duo combo device (Standard Diagnostic Inc., Korea). Secondary DENV infections were detected using Panbio Dengue IgG Capture ELISA, which has incorporated a cut-off value of > 22 Panbio Units, equivalent to HAI level of 1:2560, indicative of secondary infections [18].

Clinical data was retrospectively collected by reviewing the medical case notes, microbiology, haematology and biochemical laboratory results. The clinical data retrieved on admission included demography, vital signs, underlying comorbidities, signs and symptoms, haematological, liver and renal function parameters. Warning signs and severe dengue manifestations were recorded throughout the hospital stay. In addition, nadir platelet counts and results of dengue serology were also noted.

Approval was obtained from the Medical Research Ethics Committee, Ministry of Health Malaysia (NMRR-14-617-21061). Informed consent was not obtained from the patients, as this was a retrospective study and data was analyzed anonymously.

In total 290 patients (non-duplicate) with NS1 antigen positive were identified. Their serum samples were stored at -80 °C for further testing. These tests were conducted at an infectious diseases research laboratory at Monash University Malaysia.

### Patients’ serum samples and extraction of viral DNA

Viral RNA was extracted from 200 μl of the original serum using QIAamp viral RNA Mini Kit (Qiagen, Germany) according to the manufacturer’s instructions. Extracted RNA was stored either at -80 °C or used for RT-PCR immediately. Complementary DNA (cDNA) from viral RNA was synthesized by reverse transcription using AccessQuick RT-PCR System kit (Promega, USA). The RT mixture consisted of 10 μl (20–50 ng) of extracted RNA, 1 unit of reverse transcriptase enzyme, 12.5 μl of AccessQuick mastermix (2x), 1 μl of random primer and 20 U of RNase inhibitor (RNaseOUT, Invitrogen) in a final volume of 20 μl. The RT mixture was incubated at 65 °C for 5 min (min) followed by 37 °C for 1 h (h) and 72 °C for 5 min. The prepared cDNA was used for multiplex PCR.

### Multiplex PCR

DENV serotypes were determined using multiplex PCR [19], which amplified specific target regions using a forward conserved 5’UTR primer and four reverse primers targeting specific regions of the M and C genes of respective DENV-1, -2, -3 and -4 serotypes. To ensure the specificity of the primers to DENV and the absence of cross-reactivity with related flaviviruses, the primers were blasted through the National Centre for Biotechnology Information database [19]. Amplifications were performed as described and the expected size of each of the amplicons was as follows: DENV-1:342 bps, DENV-2: 251 bps, DENV-3: 538 bps and DENV-4: 754 bps. To perform PCR, a primer mix was prepared by mixing 400 nM of forward conserved primer and 200 nM of each reversed primer with appropriate volume of DEPC-treated distilled water. The premix was added to PCR buffer containing 1.5 mM MgCl_2,_ 0.2 mM of each of the dNTPs, 5U of Taq polymerase and 2 μl of viral cDNA. The thermal cycling profile of this assay consisted of 35 cycles of PCR at 95 °C denaturation for 30 s (s), 60 °C of annealing for 30 s and 72 °C extension for 1 min [19]. PCR contamination was avoided by spatially separating the RNA extraction, cDNA preparation and amplification steps. In order to detect possible contamination, a no template negative control was incorporated in all the PCR reactions.

All samples were also subjected to RT-PCR for Chikungunya virus based on its non-structural protein 1 (nsP1) and glycoprotein E1 (E1) genes [20]. Chikungunya virus infections are relatively common in Malaysia and can mimic DENV infections in clinical presentations. PCR was performed in a Mastercycler gradient machine (Eppendorf, Hamburg, Germany).

### Gel elution and sequencing of amplicons

The detection and identification of DENV direct from serum samples by RT-PCR was accomplished based on the product size of the amplified-serotype specific amplicons by electrophoreses in a 1.5-2 % agarose gel stained with ethidium bromide. PCR products were cut from the gel, extracted using the QIAquick Gel Extraction kit (Qiagen, Germany) and were directly sequenced in both forward and reverse directions using the specific primers by a commercial sequencing services (First base, Singapore). Random amplicons of DENVs (DENV-1: 30, DENV-2: 30, DENV-3: 13 and DENV-4:1) were selected from the PCR reactions that showed both single and dual DENV infections. The identities of the sequences were confirmed by Basic Local Alignment Search Tool (BLAST). The sequences obtained in the present study and other sequences retrieved from the GenBank were aligned in ClustalW (2.1).

### Virus propagation in C6/36 cells and total viral RNA extraction for next generation sequencing (NGS)

Confluent *Aedes albopictus* C6/36 monolayer cells were grown and maintained in minimum essential medium (MEM) supplemented with 2 % fetal bovine serum (FBS), HEPES buffer and 1 % penicillin/streptomycin (100 U/mL penicillin, 100 μg/mL streptomycin; Gibco®; USA). Virus isolation was performed by inoculating 50 μl of original serum onto C6/36 monolayer cells in Leighton tubes which were incubated at 30 °C for 7 to 10 days for growth of viruses. Viral RNA was extracted from 200 μl of the first blind passage of the serum-infected C6/36 culture supernatant and cDNA was synthesized using the method described above. The cDNA was used for multiplex PCR [19] and NGS. The amplicons derived from the multiplex PCR of supernatant of the first passage of the C6/36 infected cells were compared with those derived directly from serum. To confirm the DENV serotypes and to determine the heterogeneity of these viruses, random samples of five DENV-1 and six DENV-2 from mono-infected samples were subjected to NGS.

### Whole genome sequencing of DENV

Synthesized cDNA was converted into double stranded DNA using NEBNext® mRNA Second Strand Synthesis Module (New England Biolabs, Ipwich, MA) according to the manufacturer’s instructions. The reaction was purified using Ampure bead XP (0.8× vol. ratio), normalized to 0.2 ng/uL based on Qubit quantification (Invitrogen, Carlsbad, CA) and tagmented with Nextera XT (Illumina, San Diego, CA) according to the manufacturer’s instructions for small insert size library. The constructed libraries were quantified, normalized and sequenced on the MiSeq sequencer located at the Monash University Malaysia Genomics Facility (run configuration of 2 × 150 bps paired-end read). Reference mapping to the complete genome of DENV was performed using MITObim version 1.8 (default setting) [21]. The assembled genomes of 6 DENV-2 and 5 DENV-1 (DENV-2: TM26, TM78, TM181, TM198, TM213, TM296; DENV-1: TM24, TM50, TM99, TM100, TM242) along with additional closely related genomes of DENV isolated from the South East Asia and Oceania regions were used to infer evolutionary relationship. Nucleotide alignment based whole genome sequence was performed using MAFFT v7.127b (default alignment setting) and a maximum likelihood phylogenetic tree was constructed using FastTree version 2.1.8 with the Jukes-Cantor + CAT model [22, 23]. Tree visualization and editing was performed using FigTree v1.4.1 (http://tree.bio.ed.ac.uk/software/figtree/). Further classification of genotypes in each serotype was determined using the Genotype Determination and Recombination Detection tool on Virus Pathogen Resource (http://www.viprbrc.org/brc/genotypeRecombination.spg?method=ShowCleanInputPage&decorator=flavi_dengue).

### Definition

Warning signs (WS) assessed included abdominal pain or tenderness, persistent vomiting (≥2 consecutive days), clinical fluid accumulation, mucosal bleeding, hepatomegaly (>2 cm) and haematocrit rise concurrent with a rapid decrease in platelet counts [4]. We chose to exclude lethargy as a WS due to ambiguity in patients’ perception of lethargy and lack of objective distinction from tiredness [24]. The definition for severe dengue was obtained from the WHO 2009 criteria [4] with minor modifications and comprised at least one of the three criteria:

- Severe plasma leakage leading to shock (narrowing of pulse pressure to ≤ 20 mmHg, systolic blood pressure < 90 mm Hg or the presence of signs of poor capillary perfusion such as cold extremities, poor capillary refill or tachycardia) [3, 4] or fluid accumulation with respiratory distress (respiratory rate ≥30/min with oxygen saturation ≤ 92 % on room air, or requiring mechanical ventilation) [25].
- Severe bleeding was defined as bleeding with hemodynamic instability that requires fluid replacement for shock and/or whole blood or packed cell transfusion or any life threatening bleed, e.g. haematemesis, melaena or intracranial bleed [25].
- Severe organ impairment comprised severe liver impairment (aspartate aminotransferase or alanine aminotransferase ≥1000 IU/L), encephalopathy, myocarditis [4] or acute renal impairment (Stage 2 Acute Kidney Injury) [26, 27].

Based on population background study conducted in Malaysia, the haematocrit parameters used to evaluate haemoconcentration were >40 % in female adults, > 46 % in male ≤ 60 years, > 42 % in male > 60 years and > 38 % in children [28]. Leukopenia was defined as leukocyte count < 4,000/mm^3^ and thrombocytopenia as platelet count <150,000/mm^3^. Severe thrombocytopenia was referred to as platelet count < 50,000/mm^3^, a value shown to be associated with additional severe manifestations [7]. Paediatric patients were defined as patients aged less than 18 years. Secondary DENV infections categorization was based on the results of Panbio dengue IgG capture ELISA [18]. Pleural effusion or ascites was diagnosed based on conventional x-rays or ultrasound of the thorax and abdominal region. Diarrhoea was defined as the passage of three or more loose stools per day [28]. The simultaneous detection of more than one DENV serotypes was classified as co-infection, in contrast to mono-infection where only one DENV serotype was identified.

Data was analyzed using the Statistical Package for Social Sciences (SPSS version 20.0); comparing patients with and without DENV co-infections. To further pinpoint the variances in clinical and laboratory findings attributable to a particular serotype and its co-infection, subgroup analysis (DENV-1 and DENV-2 with its respective co-infection) was performed. Similar analysis was not conducted for DENV-3 and DENV-4 as the numbers were too small for valid statistical comparison.

Categorical variables were expressed as numbers and percentages and comparison amongst variables was determined by the Fisher’s exact test or Chi-squared test. Continuous variables were expressed as median ± interquartile range (IQR) and comparison was made using the non-parametric Mann–Whitney test. The odds ratio (OR) and its 95 % confidence intervals (CI) were calculated. The *p*-value < 0.05 (two-tailed) was taken as the level of significance. We then performed a multivariate logistic regression analysis by including clinical manifestations and laboratory parameters which were significant in univariate analysis (*P* < 0.05), to evaluate the factors independently associated with co-infections. To obtain more reliable results, variables with more than 5 % of missing data were excluded from the final model.

## Results

### Serotype distribution and phylogenetic analysis of DENV

In total 290 non-duplicate NS1 antigen positive serum samples were identified during the study period. DENV serotypes were determined by multiplex RT-PCR directly from original serum samples and from the supernatant of the first passage of C6/36 serum infected cells. The results showed the amplicons generated from both the multiplex RT-PCR were consistent with each other. Ten of the 290 samples were PCR negative for DENV. Based on the primer designs [19] these 10 samples were also negative for other flaviviruses. All the 290 samples were negative for Chikungunya virus. Of the remaining 280 samples, single DENV serotypes indicating mono-infection were detected in 238 (85 %) samples, while dual serotypes indicating co-infection were found in 42 (15 %) samples.

Medical notes for 262 of 280 patients were available for analysis (not traceable; *n* = 8; incomplete; *n* = 6; transferred out; *n* = 4). Details on DENV serotypes, demography and comorbidities are presented in Table 1. Two hundred twenty-two patients (84.7 %) were infected with a single DENV serotype and 40 patients (15.3 %) had DENV co-infections. Amongst the mono-infections, DENV- 1 (76.6 %) was by far the most common serotype identified followed by DENV- 2 (19.8 %). Seven DENV-3 were identified, and only one DENV- 4 was identified. Amongst the co-infected patients, the predominant combinations were DENV-1/DENV-2 (85 %), followed by DENV-1/DENV-3 (12.5 %) and DENV-2/DENV-3 (2.5 %). Secondary dengue infections occurred in 31.3 % of the cases.

**Table 1:** Serotype distribution, demography and comorbidities of DENV infected patients

Characteristics	*N* (%)
DENV serotypes	
Mono-infection	222 (84.7)
DENV-1	170 (76.6)
DENV-2	44 (19.8)
DENV-3	7 (3.2)
DENV-4	1 (0.5)
Co-infection	40 (15.3)
DENV-1/DENV-2	34 (85)
DENV-1/DENV-3	5 (12.5)
DENV-2/DENV-3	1 (2.5)
Race	
Malay	157 (59.9)
Chinese	34 (13)
Indian	29 (11.1)
Foreign workers	37 (14.1)
Others	5 (1.9)
Male	146 (55.7)
Age groups	
<18	38 (14.5)
18-29	104 (39.7)
30-39	64 (24.4)
40-49	27 (10.3)
50-59	17 (6.5)
>60	12 (4.6)
Pregnancy	14 (5.3)
Comorbidities^a^	52 (19.8)
Diabetes mellitus	21 (8)
Hypertension	15 (5.7)
Asthma	14 (5.3)
Cardiovascular disease	6 (2.3)
Blood disorder^b^	4 (1.5)
Malignancy^c^	4 (1.5)
Psychiatric disorders	4 (1.5)
Others^d^	5 (1.9)

^a^A patient may have more than one comorbidities

^b^Includes G6PD deficiency (*n* = 2), beta-thalassaemia (*n* = 1), von Willebrand disease (*n* = 1)

^c^Includes cancer of breast (*n* = 1), ear (*n* = 1), pancreas (*n* = 1), ovary (*n* = 1)

^d^One patient each had- chronic obstructive airway disease, military tuberculosis, splenectomy, end stage renal failure, systemic lupus erythematosus

Sequencing of representative amplicons from the mono and co-infected samples confirmed the serotype of each of the DENV. The consensus sequence of DENV-1 amplicon of size 342 bps, DENV-2 of 251 bps and DENV-3 of 538 bps were 95 % to 100 % similar for all the randomly selected 30 amplicons of DENV-1, 30 amplicons of DENV-2 and 13 amplicons of DENV-3 respectively.

**Fig. 1:**
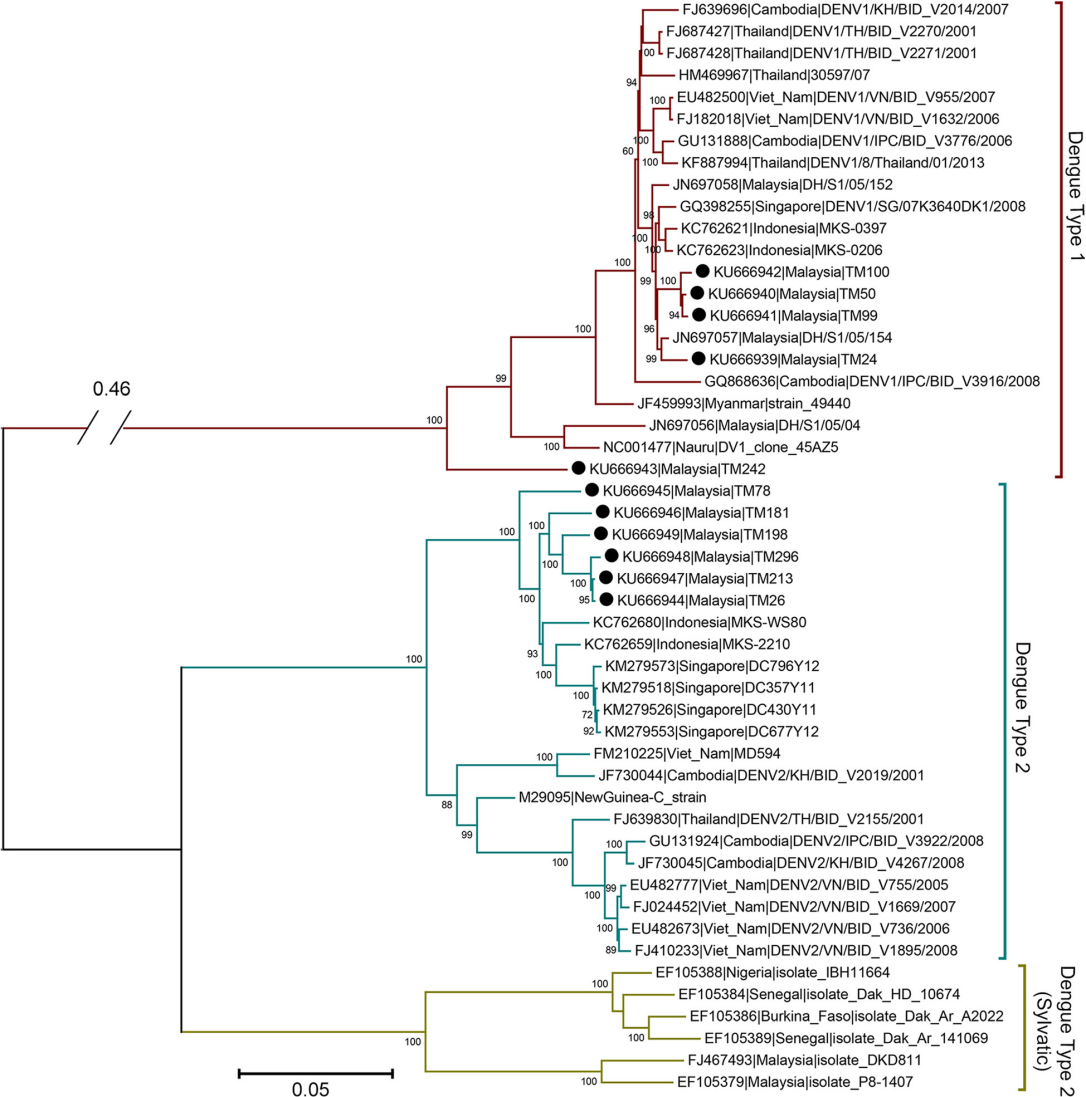
Maximum likelihood phylogeny of DENV-1 and DENV-2 strains from South East Asia and Oceania. The tree was rooted against DENV-1 (Maroon branches). Filled black circles in front of taxon names indicate strains that were sequenced and reported in this study. Taxon names are abbreviated by their Genbank accession number followed by country and strain name. Values at nodes indicate bootstrap support. Genotype classification within serotype is indicated by genotype name beside each group, within a coloured-region. Genotype I, IV and V in DENV1; genotype Cosmopolitan, Asian American (A/A), Asian-II and Asian-I in DENV-2. For clarity, the branch leading to DENV-1 has been shortened and indicated with the real length. (Scale bar: average number of substitutions per site)

Phylogenetic analysis based on whole genome sequence indicates some degrees of genomic heterogeneity among the DENV strains as evidenced by the terminal branch length in the maximum likelihood tree. The constructed phylogenomic tree exhibited the expected clustering of viral sequences based on their serotypes and genotypes (Fig. 1). All six DENV-2 isolates reported in this study fall within the same Cosmopolitan genotype and also share a common ancestry with strong bootstrap support (100 %) to DENV-2 virus isolated from regions located south of Peninsular Malaysia e.g. Singapore and Indonesia. This pattern of monophyletic clustering is also observed with the newly isolated DENV-1 virus but with a lower bootstrap support (60 %) in the DENV-1 clade with the exception of strain TM242, which is basal to the rest of the DENV-1 strains included in the phylogenomic analysis. Additionally, only TM242 has a different genotype-V while the other four reported DENV-1 strains are genotype-I. In both DENV-1 and DENV-2 lineages, the viral isolates from northern Southeast Asia countries such as Thailand, Cambodia and Vietnam are sister taxa to all or most viral isolates from Malaysia, Singapore and Indonesia, suggesting correlation with biogeography.

### Demography and comorbidities

Baseline demographic and comorbidity data is shown in Table 1. The patients’ age ranged from 3 to 75 years (median 27.75 years). There were 38 (14.5 %) paediatric patients. Majority of the patients were Malays (59.9 %). Excluding 14 pregnancies, one-fifth of patients had at least one pre-existing comorbidity; diabetes mellitus being the commonest.

### The impact of DENV co-infection on clinical and laboratory parameters on admission

Overall, the most commonly reported symptoms on admission were fever (99.2 %), vomiting (62.6 %), myalgia (59.5 %) and arthralgia (56.9 %). Particularly noteworthy was the presence of diarrhoea in almost half the patients (45.8 %). The median (IQR) duration of symptoms before hospitalization was 4 (1 to 10) days. Raised haematocrit, severe thrombocytopenia (platelets < 50,000/mm^3^) and leukopenia on admission were detected in 54.2 %, 15 % and 66.8 % of patients respectively. A substantial proportion of patients had elevated liver enzymes; 78.2 % and 61 % for AST and ALT respectively, with levels more than 10 times the normal upper limit noted in 5.5 % of patients. Elevated creatinine levels were observed in 13.5 % of patients. However, except for two patients, one with pre-existing renal failure, the creatinine levels were within 50 % of the upper limits of normal.

Univariate analysis of the comparison of clinical and laboratory findings upon admission between patients with and without DENV co-infection are shown in Tables 2 and 3. Overall, the following clinical symptoms and laboratory results on admission were significantly different (*p* < 0.05) among patients who developed DENV co-infections compared with those with mono-infections: diarrhoea (OR: 0.39; 95 % C1:0.19-0.83), fever (OR: 1.18; 95 % C1:1.12-1.25) and elevated creatinine (OR: 2.87; 95 % C1:1.23-6.70); with the latter two findings higher in the co-infected group. Similarly, on sub-group analysis, the DENV-1 co-infected patients reported diarrhoea less frequently (OR: 0.37; 95 % C1: 0.17-0.81) and were more frequently febrile (OR: 1.23; 95 % C1: 1.15-1.31) and exhibited elevated creatinine more often (OR: 2.80; 95 % C1:1.17-6.72). DENV-2 co-infected patients had a lower number of patients presenting with myalgia (OR: 0.35; 95 % C1: 0.13-0.92), arthralgia (OR: 0.34; 95 % C1: 0.13-0.93) and diarrhoea (OR: 0.32; 95 % C1: 0.12-0.83) and a higher frequency of fever (OR: 1.81; 95 % C1: 1.49-2.22) compared to mono-infected patients. In addition, severe thrombocytopenia was significantly higher in the DENV-2 co-infected group (OR: 10.75; 95 % C1: 1.25-92.16). DENV-2 co-infected patients also had significantly lower nadir platelet counts compared to the mono-infected group (*p* = 0.017).

**Table 2:** Clinical variables at time of hospital admission: comparison between DENV co-infections and mono-infections

Characteristic	All patients (*n* = 262) (%)	All mono-infections (*n* = 222) (%)	All co-infections^a^ (*n* = 40) (%)	OR (95 % CI)	DENV-1 mono-infections (*n* = 170) (%)	DENV-1 co-infections^b^ (*n* = 39) (%)	OR (95 % CI)	DENV-2 mono-infections (*n* = 44) (%)	DENV-2 co-infections^c^ (*n* = 35) (%)	OR (95 % CI)
Secondary	82 (31.3)	70 (31.5)	12 (30)	0.931 (0.45-1.94)	59 (34.7)	12 (30.8)	0.84 (0.4-1.8)	10 (22.7)	8 (22.9)	1.01 (0.35-2.9)
Pediatric	38 (14.5)	30 (13.5)	8(20)	1.6 (0.67-3.80)	23 (13.5)	8 (20.5)	1.65 (0.68-4.03)	6 (13.6)	7 (20)	1.58 (0.48-5.23)
Age > 55 years	18 (6.9)	14 (6.3)	4 (10)	1.65 (0.51-5.30)	12 (7.1)	4 (10.3)	1.51 (0.46-4.94)	1 (2.3)	3 (8.6)	4.03 (0.40-40.57)
Male	146 (55.7)	123 (55.4)	23 (57.5)	1.09 (0.55-2.15)	89 (52.4)	22 (56.4)	1.18 (0.58-2.37)	29 (65.9)	21 (60.0)	0.78 (0.31-1.95)
Pregnancy	14 (5.3)	12 (5.4)	2 (5)	0.92 (0.2-4.28)	9 (5.3)	2 (5.1)	0.97 (0.20-4.66)	2 (4.5)	2 (5.7)	1.27 (0.17-9.52)
Comorbidity	52 (19.8)	45 (20.3)	7 (17.5)	0.83 (0.35-2.01)	38 (22.4)	6 (15.4)	0.63 (0.25-1.62)	7 (15.9)	6 (17.1)	1.09 (0.33-3.61)
Fever	260 (99.2)	220 (99.1)	40 (100)	**1.18 (1.12-1.25)**	169 (99.4)	39 (100)	**1.23 (1.15-1.31)**	43 (97.7)	35 (100)	**1.81 (1.49-2.22)**
Chills and rigors	117 (44.7)	101 (45.5)	16 (40)	0.80 (0.40-1.59)	78 (45.9)	15 (38.5)	0.74 (0.36-1.50)	18 (40.9)	15 (42.9)	1.08 (0.44-2.66)
Headache	125 (47.7)	102 (45.9)	23 (57.5)	1.59 (0.81-3.14)	78 (45.9)	22 (56.4)	1.53 (0.76-3.08)	20 (45.5)	20 (57.1)	1.6 (0.65-3.91)
Cough	30 (11.5)	28 (12.6)	2 (5)	0.37 (0.08-1.60)	20 (11.8)	2 (5.1)	0.41 (0.09-1.81)	8 (18.2)	2 (5.7)	0.27 (0.05-1.38)
Nausea	72 (27.5)	59 (26.6)	13 (32.5)	1.33 (0.64-2.75)	41 (24.1)	13 (33.3)	1.57 (0.74-3.34)	15 (34.1)	10 (28.6)	0.77 (0.30-2.03)
Vomit	164 (62.6)	141 (63.5)	23 (57.5)	0.78 (0.39-1.54)	111 (65.3)	22 (56.4)	0.69 (0.34-1.40)	26 (59.1)	20 (57.1)	0.92 (0.38-2.2)
Anorexia	101 (38.7)	83 (37.4)	18 (45)	1.37 (0.69-2.70)	61 (35.9)	18 (46.2)	1.53 (0.76-3.09)	21 (47.7)	16 (45.7)	0.92 (0.38-2.25)
Abdominal pain	103 (39.3)	91 (41.0)	12 (30)	0.62 (0.30-1.28)	73 (42.9)	12 (30.8)	0.59 (0.28-1.24)	16 (36.4)	9 (25.7)	0.61 (0.23-1.61)
Diarrhoea	120 (45.8)	109 (49.1)	11 (27.5)	0.39 (0.19-0.83)*	82 (48.2)	10 (25.9)	0.37 (0.17-0.81)*	23 (52.3)	9 (25.7)	0.32 (0.12-0.83)*
Myalgia	156 (59.5)	134 (60.4)	22 (55)	0.80 (0.41-1.58)	95 (55.9)	21 (53.8)	0.92 (0.46-1.85)	34 (77.3)	19 (54.3)	0.35 (0.13-0.92)*
Arthralgia	149 (56.9)	128 (57.7)	21 (52.5)	0.81 (0.41-1.60)	90 (52.9)	20 (51.3)	0.94 (0.47-1.88)	35 (79.5)	20 (57.1)	0.34 (0.13-0.93)*
Rash	39 (14.9)	35 (15.8)	4 (10)	0.59 (0.20-1.77)	23 (13.5)	4 (10.3)	0.73 (0.24-2.25)	11 (25)	4 (11.4)	0.39 (0.11-1.34)
Neurological	20 (7.6)	17(7.7)	3 (7.5)	0.98 (0.27-3.50)	14 (8.2)	3 (7.7)	0.93 (0.25-3.40)	2 (4.5)	2 (5.7)	1.27 (0.17-9.52)
Haemorrhagic symptoms	45 (17.2)	40(18.0)	5 (12.5)	0.65 (0.24-1.76)	34 (20)	5 (12.8)	0.59 (0.21-1.62)	2 (4.5)	4 (11.4)	2.71 (0.47-15.75)
Documented fever	189 (72.1)	159(71.6)	30 (75)	1.19 (0.55-2.58)	120 (70.6)	29 (74.4)	1.21 (0.55-2.67)	31 (70.5)	25 (71.4)	1.05 (0.39-2.79)
Tachypnoea	32 (12.3)	28(12.6)	4 (10)	0.77 (0.26-2.33)	20 (11.8)	4 (10.3)	0.86 (0.28-2.67)	8 (18.2)	4 (11.4)	0.58 (0.16-2.12)
Tachycardia	64 (24.4)	53(23.9)	11 (27.5)	1.21 (0.57-2.59)	47 (27.6)	11 (28.2)	1.03 (0.47-2.23)	6 (13.6)	10 (28.6)	2.53 (0.82-7.85)
Hypotension	22 (8.4)	20(9)	2 (5)	0.53 (0.12-2.37)	17 (10)	2 (5.1)	0.49 (0.11-2.20)	3 (6.8)	1 (2.9)	0.40 (0.04-4.04)

Bold* type represents significance at *p* < 0.05

^a^DENV-1/DENV-2 (*n* = 34), DENV-1/DENV-3 (*n* = 5), DENV-2/DENV-3 (*n* = 1); ^b^DENV-1/DENV-2 (*n* = 34), DENV-1/DENV-3 (*n* = 5); ^c^DENV-2/DENV-1 (*n* = 34), DENV-2/DENV-3 (*n* = 1)

**Table 3:** Laboratory variables at time of hospital admission: comparison between DENV co-infections and mono-infections

Characteristic	All patients (*n* = 262) (%)	All mono-infections (*n* = 222) (%)	All co-infections^a^ (*n* = 40) (%)	OR (95 % CI)	DENV-1 mono-infections (*n* = 170) (%)	DENV-1 co-infections^b^ (*n* = 39) (%)	OR (95 % CI)	DENV-2 mono-infections (*n* = 44) (%)	DENV-2 co-infections^c^ (*n* = 35) (%)	OR (95 % CI)
Leukopenia	175 (66.8)	149 (67.1)	26 (65)	0.91 (0.45-1.85)	121(71.2)	25 (64.1)	0.72 (0.35-1.51)	21 (47.7)	21 (60)	1.64 (0.67-4.04)
Platelet < 50,000/mm^3^	39 (14.9)	30 (13.5)	9 (22.5)	1.86 (0.81-4.29)	27(15.9)	9 (23.1)	1.59 (0.68-3.72)	1 (2.3)	7 (20)	10.75 (1.25-92.16)*
Raised haematocrit	142 (54.2)	117 (52.7)	25 (62.5)	1.50 (0.75-2.99)	88(51.8)	23 (59)	1.34 (0.66-2.71)	23 (52.3)	23 (65.7)	1.75 (0.7-4.37)
Raised urea^d^	11 (4.6)	9 (4.5)	2 ( 5.4)	1.22( 0.25-5.88)	7(4.5)	2 (5.6)	1.25 (0.25-6.30)	2 (5.4)	2 (6.2)	1.17 (0.16-8.79)
Low sodium	9 (3.7)	7 (3.4)	2 (5.3)	1.58 (0.32-7.91)	6(3.8)	2 (5.4)	1.45 (0.28-7.48)	1 (2.5)	2 (6.1)	2.52 (0.22-29.05)
Low potassium	168 (68.9)	145 (70.4)	23 (60.5)	0.65 (0.32-1.32)	108(68.4)	22 (59.5)	0.68 (0.33-1.42)	31 (77.5)	21 (63.6)	0.51 (0.18-1.42)
Raised creatinine^e^	32 (13.5)	22 (11.1)	10 (26.3)	2.87 (1.23-6.70)*	18(11.7)	10 (27)	2.80 (1.17-6.72)*	4 (10.8)	9 (27.3)	3.09 (0.85-11.24)
Raised bilirubin	12 (4.8)	10 (4.7)	2 (5.3)	1.12 (0.24-5.31)	9(5.6)	2 (5.4)	0.97 (0.2-4.67)	1 (2.4)	2 (6.1)	2.65 (0.23-30.51)
Low albumin	7 (2.8)	6 (2.8)	1 (2.6)	0.94 (0.11-8.01)	4(2.4)	1 (2.7)	1.11 (0.12-10.24)	2 (4.8)	1 (3)	0.63 (0.05-7.21)
Raised AST^f^	133 (78.2)	114 (78.6)	19 (76)	0.86 (0.32-2.34)	90(81.1)	19 (79.2)	0.89 (0.30-2.65)	19 (65.5)	17 (77.3)	1.79 (0.51-6.29)
Raised ALT	153 (61)	133 (62.4)	20 (52.6)	0.67 (0.33-1.34)	108(65.9)	20 (54.1)	0.61 (0.30-1.26)	20 (47.6)	19 (57.6)	1.49 (0.60-3.74)

Bold* type represents significance at *p* < 0.05

^a^DENV-1/DENV-2 (*n* = 34), DENV-1/DENV-3 (*n* = 5), DENV-2/DENV-3 (*n* = 1); ^b^DENV-1/DENV-2 (*n* = 34), DENV-1/DENV-3 (*n* = 5); ^c^DENV-2/DENV-1 (*n* = 34), DENV-2/DENV-3 (*n* = 1)

^d^Data available for 238 cases, ^e^Data available for 237 cases, ^f^Data available for 170 cases

Multivariate analysis showed that the following clinical findings and laboratory results were independently associated with co-infections. Overall, diarrhoea was negatively associated with co-infections (OR: 0.326; 95 % CI: 0.149-0.711). Likewise, diarrhoea was less common in DENV-1 (OR: 0.339; 95 % CI: 0.151-0.764) and DENV-2 co-infections (OR: 0.24; 95 % CI: 0.077-0.753), compared to the mono-infected group. In addition, DENV-2 co-infected group had a higher frequency of patients with severe thrombocytopenia on admission (OR: 12.561; 95 % CI: 1.297-121.647), whereas the DENV-2 mono-infected group had a higher number of patients manifesting with myalgia (OR: 0.306; 95 % CI: 0.102-0.919). Although elevated creatinine levels were significantly higher in the co-infected group in univariate analysis, it was not subjected to multivariate analysis, as missing values were > 5 %.

Comorbidities and pregnancies were not associated with co-infection, both in overall and sub-group analysis. Likewise, no significant association with co-infection was elicited when each comorbidity (Table 1) was analyzed separately. Co-infection was not related to gender, ethnicity, age and secondary DENV infections. Similarly, there was no significant difference in the frequency of haemorrhagic manifestations and raised haematocrit between the two groups.

### The impact of DENV co-infection on disease severity

The comparison of disease severity between patients with mono-infection and co-infection is shown in Table 4. Majority (78 %) of patients presented with at least one warning sign. Amongst the spectrum of warning signs, abdominal pain/tenderness (45.4 %) and persistent vomiting (43.5 %) were the two most common*.* In total, 17 patients (6.5 %) presented with severe dengue manifestations: these were fluid accumulation with respiratory distress (*n* = 7), shock (*n* = 4), severe bleeding (*n* = 2), and severe organ impairment (*n* = 9). The organs involved were liver (*n* = 4), central nervous system (*n* = 4) and renal (*n* = 1). The DENV serotypes associated with severe dengue were: DENV-1 (*n* = 9), DENV-2 (*n* = 1), DENV-3 (*n* = 1) in mono-infected patients and DENV-1/DENV-2 (*n* = 5) and DENV-1/DENV-3 (*n* = 1) amongst those with co-infection. There was one fatality recorded, involving a 28-year-old lady at day 46 post-partum. She was transferred from another district hospital and presented late in illness when she was already in shock. She had secondary dengue infection and was infected with DENV-1.

**Table 4:** Clinical characteristics based on disease severity^a^: Comparison between DENV co-infections and mono-infections

Characteristic	All patients (*n* = 262) (%)	All mono-infections (*n* = 222) (%)	All co-infections^b^ (*n* = 40) (%)	OR (95 % CI)	DENV-1 mono-infections (*n* = 170) (%)	DENV-1 co-infections^c^ (*n* = 39) (%)	OR (95 % CI)	DENV-2 mono-infections (*n* = 44) (%)	DENV-2 co-infections^d^ (*n* = 35) (%)	OR (95 % CI)
Any warning signs	204 (77.9)	168 (75.7)	36 (90)	2.89 (0.99-8.50)*	134 (78.8)	35 (89.7)	2.35 (0.78-7.05)	29 (65.9)	32 (91.4)	5.52 (1.45-21.02)*
Persistent vomiting	114 (43.5)	99 (44.6)	15 (37.5)	0.75 (0.37-1.49)	82 (48.2)	15 (38.5)	0.67 (0.33-1.37)	14 (31.8)	13 (37.1)	1.27 (0.50-3.22)
Abdominal pain/tenderness	119 (45.4)	100 (45)	19 (47.5)	1.10 (0.56-2.17)	81 (47.6)	19 (48.7)	1.04 (0.52-2.09)	17 (38.6)	16 (45.7)	1.34 (0.54-3.29)
Mucosal bleeding	64 (24.4)	52 (23.4)	12 (30)	1.40 (0.67-2.95)	38 (22.4)	12 (30.8)	1.54 (0.72-3.33)	10 (22.7)	12 (34.3)	1.77 (0.66-4.78)
Tender hepatomegaly	9 (3.4)	7 (3.2)	2 (5)	1.62 (0.32-8.08)	3 (1.8)	2 (5.1)	3.01 (0.49-18.65)	4(9.1)	2 (5.7)	0.61 (0.10-3.52)
Pleural effusion	6 (2.3)	2 (0.9)	4 (10)	12.22 (2.16-69.19)*	2 (1.2)	4 (10.3)	9.6 (1.69-54.48)*	0	4 (11.4)	OR Undefined* **(** ***p*** **= 0.035)**
Increasing haematocrit with decreasing platelets	64 (24.4)	52 (23.4)	12 (30)	1.40 (0.67-2.95)	41 (24.1)	11 (28.2)	1.24 (0.57-2.70)	10 (22.7)	11 (31.4)	1.56 (0.57-4.25)
Any severe dengue manifestations	17 (6.5)	11 (5)	6 (15)	3.39 (1.174-9.76)*	9 (5.3)	6 (15.4)	3.25 (1.08-9.76)*	1 (2.3)	5 (14.3)	7.17 (0.80-64.49)
Shock	4 (1.5)	3 (1.4)	1 (2.5)	1.87 (0.19-18.46)	2 (1.2)	1 (2.6)	2.21 (0.20-25.01)	1 (2.3)	0	OR Undefined
Fluid accumulation with respiratory distress	7 (2.7)	3 (1.4)	4 (10)	8.11 (1.74-37.76)*	3 (1.8)	4 (10.3)	6.36 (1.36-29.70)*	0	4 (11.4)	OR Undefined* **(** ***p*** **= 0.035)**
Severe bleeding	2 (0.8)	2 (0.9)	0	OR Undefined	1 (0.6)	0	OR Undefined	0	0	NA
Severe organ involvement	9 (3.4)	7 (3.2)	2 (5.0)	1.62 (0.32-8.08)	7 (4.1)	2 (5.1)	1.26 (0.25-6.31)	0	2 (5.7)	OR Undefined
ICU	4 (1.5)	4 (1.8)	0	OR Undefined	4 (2.4)	0	OR Undefined	0	0	NA
Oxygen supplementation	7 (2.7)	5 (2.3)	2 (5)	2.28 (0.43-12.20)	5 (2.9)	2 (5.1)	1.78 (0.33-9.55)	0	2 (5.7)	OR Undefined
Mechanical ventilation	4 (1.5)	4 (1.8)	0	OR Undefined	4 (2.4)	0	OR Undefined	0	0	NA
Hospitalization > 3 days	96 (36.6)	84 (37.8)	12 (30)	0.70 (0.34-1.46)	60 (35.3)	12 (30.8)	0.82 (0.39-1.72)	21 (47.7)	11 (31.4)	0.50 (0.20-1.27)

Bold* type represents significance at *p* < 0.05. ^a^Disease severity is based on WHO (2009) classification plus additional clinical criteria

^b^DENV-1/DENV-2 (*n* = 34), DENV-1/DENV-3 (*n* = 5), DENV-2/DENV-3 (*n* = 1); ^c^DENV-1/DENV-2 (*n* = 34), DENV-1/DENV-3 (*n* = 5); ^d^DENV-2/DENV-1 (*n* = 34), DENV-2/DENV-3 (*n* = 1),

In univariate analysis (Table 4), the presence of at least one warning sign was significantly higher amongst the co-infected group (OR: 2.89; 95 % C1: 0.99-8.50). Amongst the warning signs, co-infected patients were 12 times at increased risk of developing pleural effusion (OR: 12.22; 95 % C1: 2.16-69.19). A higher proportion of co-infected (15 %) patients had at least one severe dengue manifestation compared to mono-infected (5 %) patients (OR: 3.39; 95 % C1: 1.174-9.76). Except for severe bleeding, all the other entities of severe dengue appeared at higher frequency in the co-infected group. However, the difference was statistically significant only for fluid accumulation with respiratory distress (OR: 8.11; 95 % C1: 1.74-37.76). Compared to the mono-infected group, DENV-1 co-infected patients had a significantly higher numbers of pleural effusion (OR: 9.6; 95 % C1: 1.69-54.48), severe dengue manifestations (OR: 3.25; 95 % C1: 1.08-9.76) and fluid accumulation with respiratory distress (OR: 6.36; 95 % C1: 1.36-29.70). Warning signs, pleural effusion and fluid accumulation with respiratory distress were also significantly higher in the DENV-2 co-infected group.

Multivariate analysis revealed that pleural effusion (OR: 12.227; 95 % CI: 1.998 -74.817) and the presence of warning signs (OR: 3.143; 95 % CI: 1.047-9.429) were positively associated with co-infections. Subgroup analysis comparing DENV-2 mono and co-infections, revealed similar characteristics as above. Subgroup analysis comparing DENV-1 mono and co-infected patients, revealed that pleural effusion (OR: 11.824; 95 % CI: 1.936-72.203) was positively associated with co-infections. The associations in the multivariate analysis were maintained when adjusted for comorbidity and patients with secondary dengue infections.

We found no statistical differences between the two groups and its sub-groups in other manifestations of disease severity, such as hypotension, shock, admission to an intensive care unit, the need for mechanical ventilation or supplemental oxygen and hospitalization duration of > 3 days. The median (IQR) length of hospital stay was 3 (1–16) days for the mono-infected and 3 (1–18) days for the co-infected groups.

## Discussion

The co-circulation of multiple DENV serotypes within a similar geographical area provides a suitable niche for the occurrence of co-infections, a phenomenon best observed during epidemics [8, 10, 11, 14–17]. The first case of co-infection with 2 dengue virus serotypes (DENV-1 and DENV-4) was reported in Puerto Rico in 1982 [29]. Since then, various reports have emerged from various countries describing the occurrence of co-infections. Co*-*infection rates vary widely in different countries and different regions within the same country. In the present study, the overall co-infection rate was 15 %. These rates were comparable to those in New Delhi [10, 11], Ceylon [30] and Vietnam [12], whereas other regions such as Indo-China [9], Brazil [15], Kerala [16] and Karnataka [17] have reported higher rates. Nevertheless, very few of these studies have specifically explored the clinical impact of co-infection as the actual number of co-infected cases in these studies have been rather small. To the best of our knowledge, this study provides the most in-depth insight of the association between co-infection and the various clinical and laboratory parameters and also has the largest pool of co-infected cases.

In the present study, there was an overwhelming predominance of DENV-1, followed by DENV-2 and DENV-3, with only one patient infected with DENV-4. Correspondingly, the common co-infections involved the most common DENV serotypes, as evidenced by an overwhelming co-infections caused by DENV-1/DENV-2 (85 %) followed by DENV-1/DENV-3 (12.5 %). The results of multiplex PCR from both the original serum and first passage of the supernatant of C6/36 serum infected cells demonstrated consistency in terms of determining the serotypes of the DENV.

Phylogenetic analysis indicated that the DENV strains were not clonal and showed heterogeneity amongst them. The close relatedness of the DENV isolates as revealed by monophyletic clustering indicated local dengue outbreaks. In addition, this study significantly expanded the number of Malaysian DENV-1 whole genomes by 5-fold and to our knowledge, is the first to report the complete DENV-2 genomes from Malaysia.

It may be possible to acquire co-infection from a single mosquito bite in endemic areas where more than one serotypes are circulating, as the presence of two DENV serotypes in one mosquito has been shown [31]. Co-infection may also be possible if the patient is bitten by two different mosquitos within a short period. The chances of dual dengue infections of humans are further enhanced because of the feeding behaviour of *Aedes aegypti* [14]. Female *Ae. aegypti* feeds numerous times on human host during each gonotrophic cycle, increasing the opportunities of spreading *Ae. aegypti* borne-disease [32]. Furthermore, the time spent probing is lengthier in infected compared to uninfected *Ae. Aegypti* mosquitoes. Longer feeding periods encourages more host defensive behaviours against the blood-seeking mosquitoes, increasing the probability that an infected mosquito will probe on additional hosts [33]. This feeding behaviour facilitates dual infections in mosquitoes with subsequent transmission of multiple DENV to single human host [14].

Apart from the typical dengue-related symptoms, it was interesting to note that diarrhoea was the presenting symptom in almost half of the patients. Significantly higher frequency of diarrhoea was noted amongst the mono-infected compared with co-infected patients. Other gastrointestinal symptoms also appeared higher in the mono-infected group, although not statistically significant. Cytokines and interleukins (ILs) play a major role in the pathogenesis of dengue fever, with a possible role of IL-8 in the pathogenesis of dengue-associated diarrhoea [34]. Co-infections may result in synergistic or antagonistic interactions which may alter disease pathogenesis, thus altering the clinical presentation of disease. Another difference observed on subgroup analysis was that DENV-2 mono-infected patients were more likely to present with myalgia and arthralgia compared to co-infected patients. Conversely, in another Brazilian study, arthralgia was more common amongst the co-infected patients [8]. However, apart from gastrointestinal and musculoskeletal symptoms, we found no other individual symptoms or group of symptoms that distinguished DENV mono and co-infections.

The frequency of patients with severe thrombocytopenia on admission and lower nadir platelet counts was significantly higher amongst the DENV-2 co-infected compared to mono-infected patients. Likewise, the association between low platelets and DENV co-infection was also noted in another study [8]. While some studies revealed a higher frequency of haemorrhagic manifestations amongst DENV co-infected patients [8, 10, 17]; concurring with other studies [12, 14] we found no such association. This supports previous findings that the degree of thrombocytopenia does not necessarily correlate with haemorrhage, and other triggers such as liver injury, vasculopathy, activation of coagulation and fibrinolytic system, release of pro-inflammatory cytokines and platelet dysfunction may contribute towards dengue associated bleeding diatheses [35].

Controversy still exists as to whether the presence of co-infection increases disease severity. In the present research, the presence of warning signs (90 %) and other severe disease manifestations (15 %) were significantly higher amongst patients with DENV co-infection, concurring with a recent study in Brazil which revealed that 61 % of the co-infected patients had either severe dengue or dengue with warning signs [8]. Although pleural effusion was significantly more common amongst the co-infected patients, there was no corresponding increase in other parameters of plasma leakage such as hypoalbuminaemia or raised haematocrit. This could partially be explained by the fact that we analyzed these parameters upon admission, which could possibly alter during the course of disease. Binh PT et al. reported no increase in plasma leakage amongst DENV co-infected patients [12]. Neither was there an increased occurrence of gallbladder thickening which reflects tissue oedema resulting from plasma leakage [12].

The mechanisms of disease virulence and the resulting clinical manifestations in DENV co-infections remains largely unclear. Heterogeneity in patient characteristics, differences in patient population i.e. outpatient verses hospitalized patients, differences in DENV serotypes and variances in the parameters assessed may explain the differences found. Furthermore, the studies reported so far have involved relatively small numbers of co-infections, making statistical inferences difficult. The possible clinico- pathological effects of DENV co-infections resulting from direct interactions of viral genes or indirect interactions resulting in alterations in the host-environment or immunological changes [36], need further exploration. The presence of DENV co-infection is likely to increase DENV viremia levels [8]. Higher viremia early in the course of infection has been linked to increased disease severity and higher frequency of pleural effusion [35, 37]. Another worrying consequence of co-infections is the possible occurrence of recombination events [38], which may result in alteration of DENV virulence.

### Strengths and limitations

The strength of this study is that it involved a large number of co-infected patients, allowing more reliable data interpretation. Moreover, since blood samples were collected during the acute phase of disease, the serum samples were NS1 antigen and RT-PCR positive, which permitted serotyping and viral isolation.

However, there are several limitations to this current study. All retrospective studies depend on the obtainability, accuracy and completeness of medical data. Therefore, the influence of certain clinical findings such as petechial rash and Hess’s test were difficult to determine as these tests/findings may not have been performed or recorded in medical charts. Moreover, this study had a relatively small number of patients with pleural effusion which may result in a low statistical power to detect true association. Chest x-rays were performed at the discretion of the attending clinicians, according to clinical findings. However, chest x-rays can only detect significant pleural effusions. The use of serial chest ultrasound can improve detection of small pleural effusions [39], although this may not be practical because of budgetary constraints and service restraints. However, despite this, all patients were managed with a standardized dengue clinical care management algorithm and laboratory and clinical parameters essential for patient monitoring were carefully recorded in the medical charts, which helped improve reliability of data collected. While a prospective study would be more reliable and accurate, the practicality of such a study is questionable because of the low numbers of co-infections in most reports. Finally, our study was conducted in a tertiary referral hospital and involved only hospitalized patients. Thus, these findings may not be generalized to patients with less severe manifestations, not necessitating hospitalization.

## Conclusion

In conclusion, our findings suggest that DENV co*-*infections are not a rare occurrence and may play a previously unrecognized role in the pathogenesis, virulence and clinical expression of disease. Patients with co-infections seemed lop-sided towards more severe clinical manifestations as evidenced by a higher frequency of severe thrombocytopenia, pleural effusion, elevations of creatinine levels, and the presence of warning signs. However, haemorrhagic manifestations and haemoconcentration did not appear to be higher in the co-infected group. Co-infections may also alter clinical presentation as suggested by a lower number of diarrhoea and arthralgia/myalgia symptoms amongst these patients compared to the mono-infected patients. Although our report illuminates important finding on the impact of DENV co-infections, a prospective study performed on a larger scale will be useful to further strengthen these findings. Exploration of cellular immune response and host cytokines associated with DENV co-infections is a logical next step.
